# Comorbidities in COPD: Current and Future Treatment Challenges

**DOI:** 10.3390/jcm13030743

**Published:** 2024-01-27

**Authors:** Domenica Francesca Mariniello, Vito D’Agnano, Donatella Cennamo, Stefano Conte, Gianluca Quarcio, Luca Notizia, Raffaella Pagliaro, Angela Schiattarella, Rosario Salvi, Andrea Bianco, Fabio Perrotta

**Affiliations:** 1 Department of Translational Medical Sciences, University of Campania “L. Vanvitelli”, 80131 Naples, Italy; nikamariniello93@gmail.com (D.F.M.); vito.dagnano@studenti.unicampania.it (V.D.); donatella.cennamo@studenti.unicampania.it (D.C.); stefano.conte@unicampania.it (S.C.); gianluca.quarcio@studenti.unicampania.it (G.Q.); luca.notizia@studenti.unicampania.it (L.N.); raffaella.pagliaro@studenti.unicampania.it (R.P.); angela.schiattarella@studenti.unicampania.it (A.S.); andrea.bianco@unicampania.it (A.B.); 2 U.O.C. Chirurgia Toracica, Azienda Ospedaliera “S.G. Moscati”, 83100 Avellino, Italy; rosdoc@libero.it

**Keywords:** COPD, comorbidities, cardiovascular disease, obstructive sleep apnea, bronchiectasis, metabolic syndrome

## Abstract

Chronic obstructive pulmonary disease (COPD) is a heterogeneous lung condition, primarily characterized by the presence of a limited airflow, due to abnormalities of the airways and/or alveoli, that often coexists with other chronic diseases such as lung cancer, cardiovascular diseases, and metabolic disorders. Comorbidities are known to pose a challenge in the assessment and effective management of COPD and are also acknowledged to have an important health and economic burden. Local and systemic inflammation have been proposed as having a potential role in explaining the association between COPD and these comorbidities. Considering that the number of patients with COPD is expected to rise, understanding the mechanisms linking COPD with its comorbidities may help to identify new targets for therapeutic purposes based on multi-dimensional assessments.

## 1. Introduction

**Figure 1:**
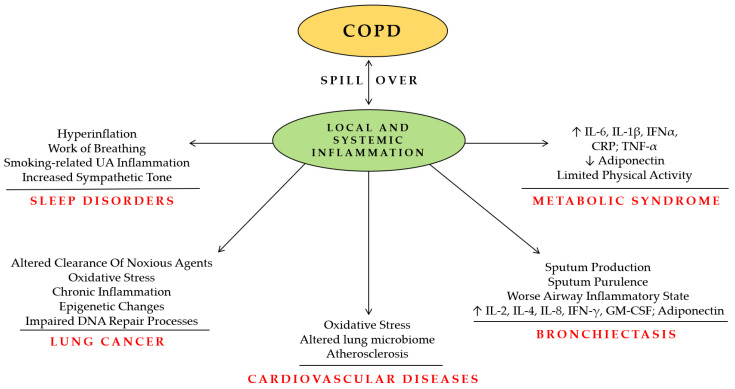
Chronic obstructive pulmonary disease (COPD) and comorbidities. COPD: Chronic obstructive pulmonary disease; IL-6: Interleukin 6; IL-1β: Interleukin-1beta; IFNα: Interferon alfa; CRP: C-reactive protein; TNFα: Tumor Necrosis Factor alfa; IL-2: Interleukin 2; IL-4: Interleukin 4; IL-8: Interleukin 8; GM-CSF: granulocyte–macrophage colony-stimulating factor (GM-CSF); ↑ increased expression; ↓ reduced expression.

Chronic obstructive pulmonary disease (COPD) is a heterogeneous lung condition characterized by chronic respiratory symptoms such as dyspnea, cough, and sputum production and is one of the major chronic respiratory conditions both in terms of morbidity and mortality worldwide. The main casual factor is prolonged exposure to cigarette smoke or other noxious particles which lead to reduced air flow and pulmonary hyperinflation. Although COPD primarily affects the lungs, it is also recognized as a complex multi-systemic disease that frequently coexists with other conditions known as comorbidities. The coexistence of concurrent disorders—including cardiovascular, metabolic, and cancer—may contribute to a worse prognosis and quality of life compared to patients without further comorbidities. Evidence suggests that the association between COPD and its comorbidities is the result of the ‘‘spill-over’’ of systemic inflammation in which Interleukin (IL)-6, IL-1β, C-reactive protein (CRP), and Tumor Necrosis Factor (TNF)-α play a primary role [1,2,3]. Other possible mechanisms that play a role in COPD and its comorbidities include common risk factors like aging, smoking, and physical inactivity, and common pathways such as oxidative stress [4]. The main aim of this review is to analyze the major comorbidities, such as other pulmonary conditions (lung cancer, bronchiectasis, and sleep disorders), cardiovascular diseases (CVDs), and metabolic disorders and their impact on patients with COPD (Figure 1). Given the impact of these comorbidities on COPD progression, mortality, and resource use, it is crucial to have proper patient management and initiate the early treatment of these conditions.

## 2. COPD and Cardiovascular Diseases

### 2.1. Pathogenesis and Mechanisms of Cardiopulmonary Interaction

Several studies have shown that patients with COPD are particularly vulnerable to cardiovascular morbidity and mortality, with a higher incidence and prevalence of CVDs than the general population [5]. Evidence suggests that mortality due to CVD is more common than those related to respiratory failure in patients with COPD [6]. Moreover, patients with the co-existence of COPD and CVD experience worse dyspnea and exercise tolerance, and a higher risk of hospitalization for COPD and for CVD [6]. The most common cardiovascular diseases in COPD are ischemic heart disease, arrhythmias, and heart failure. The mechanistic links between COPD and cardiovascular disease are complex, multifactorial, and not fully understood. Both disease share common risk factors such as cigarette smoking, aging and physical inactivity [7]. Pathophysiological links between COPD and CVD include lung hyperinflation, pulmonary hypertension, hypoxemia, oxidative stress, and systemic inflammation. Static hyperinflation is characterized by an abnormally increased volume of residual gas trapped in the lungs following spontaneous expiration, leading to airflow limitation, increased intrathoracic pressure, and heart dysfunction. Conversely, the progressive loss of inspiratory capacity during the exercise associated with an increase in end-expiratory lung volume as a result of the impaired elastic properties of lung parenchyma is named dynamic hyperinflation. In an experimental study involving healthy subjects, the increase in dynamic hyperinflation—administered through expiratory pressure—was associated with a significant drop in left ventricular stroke volume as well an increase in the end-diastole radius curvature of the interventricular septum [8]. Progressive airflow limitation and emphysema lead to a ventilation/perfusion mismatch that is the principal contributor to hypoxemia, leading to pulmonary hypertension and, consequently, to right and left heart failure.

Another shared hallmark of COPD and CVDs is low-grade systemic inflammation. In stable COPD patients, fine-tuned mechanisms including innate and adaptative activation triggered by exogenous factors have been described [9]. Low-grade inflammation seems also to promote cardiovascular diseases through (a) an increase in the formation and rupture of atherosclerotic plaques, (b) the proliferation of smooth cell leading to increased arterial stiffness, (c) the promotion of platelet aggregation, (d) exalted endothelial dysfunction, and (e) a reduction in progenitor endothelial cells (CD34+) [10]. During acute exacerbation, COPD patients are particularly susceptible to cardiovascular events. An acute exacerbation of COPD was associated with a 5 day transiently increased risk for acute myocardial infarction [11]. Cardiac biomarkers such as CRP, fibrinogen, N-terminal proBNP (NT-proBNP), and troponin are increased in patients with COPD exacerbations, and these are independent risk factors for mortality. In patients with COPD, elevated NT-pro-BNP levels are associated with a longer length of hospital stay and the need for intensive care [12,13,14]. Inflammation in COPD also promotes increased procoagulant activity. In turn, coagulation amplifies inflammation, and both are strongly implicated in the pathogenesis of atherothrombosis [15].

### 2.2. Cardiovascular Mortality in COPD Pharmacological Trials

Several researches have attempted to tackle all-cause and cardiovascular mortality. The UPLIFT study was a 4-year longitudinal study evaluating the impact of tiotropium versus placebo. The trial documented a 16% reduction in both respiratory and cardiovascular mortality (*p* = 0.016). A subsequent analysis revealed poor concordance between the study investigators and the mortality adjudication committee [16].

Two studies (TORCH and SUMMIT) [17,18] investigated the combination of ICS+LABA (Salmeterol/fluticasone propionate and vilanterol/fluticasone furoate) versus placebo. The results did not indicate advantages in mortality. Recent analyses of studies in the literature also suggest the absence of a positive impact on mortality in systematic reviews comparing LABA-LAMA combinations versus monocomponents, ICS-LABA, or placebo [19,20,21]. For triple fixed inhaled therapies (ICS-LABA-LAMA), two independent large randomized clinical trials (RCTs) have provided the proof of concept for the efficacy of these combinations on cardiovascular mortality. The IMPACT study [22] demonstrated the superiority of the triple combination (fluticasone furoate/umeclidinium/vilanterol, “FF/UMEC/VI” 100/62.5/25 mcg) compared to the two different dual-combination classes, fluticasone furoate/vilanterol (FF/VI) and umeclidinium/vilanterol (UMEC/VI), regarding the reduction in the annual rate of moderate/severe exacerbations and an improvement in lung function and quality of life. In addition, a significant 34% reduction in hospitalizations for severe COPD exacerbations was observed with the triple combination compared to umeclidinium/vilanterol, and a 13% numerical reduction was observed when compared with fluticasone furoate/vilanterol, though it was not statistically significant. A 28% reduction in the risk of mortality (mainly cardiovascular) during treatment with the triple combination was observed compared with the umeclidinium/vilanterol combination. Similarly, the ETHOS study included symptomatic COPD patients with a documented history of at least one exacerbation despite regular treatment with at least dual therapy [23]. The results of this study demonstrate that budesonide/glycopyrrolate/formoterol fumarate triple therapy statistically significantly reduced the rate of moderate or severe exacerbations compared with dual combination therapies in patients with moderate to very severe COPD. Furthermore, the combination of budesonide, glycopirronium, and formoterol reduces the all-cause mortality when compared with the glycopirronium and formoterol combination as a result of the reduction in cardiovascular events (HR 0.51, IC 0.33–0.80). While the advantages in all-cause and cardiovascular mortality is an outcome coherent with a positive impact on the exacerbation risk as well improvements in respiratory symptoms and spirometry, the results of the RCTs should be interpretated with caution as there is the potential interference of ICS withdrawal in ICS users who were randomized into the LABA-LAMA group [24].

### 2.3. Efficacy and Safety of Cardiovascular Agents in Patients with COPD

Pulmonary and cardiovascular pharmacological interactions are of leading clinical importance as common regulatory pathways are involved. Beta-blockers are the cornerstone of the treatment for many heart disease; they are often underused in COPD patients due to their potential antagonism with beta-2-agonists which leads to acute bronchospasm [25]. Based on the recent literature, however, this is considered an erroneous concept because the use of beta-blockers not only alleviates the symptoms of COPD by improving heart function but also shows beneficial effects on COPD itself, reducing mortality, hospital admission, and the risk of exacerbations [26]. European Society of Cardiology (ESC) guidelines state that COPD is not a contraindication for the use of β-blockers, but selective β_1_-blockers (e.g., atenolol, bisoprolol, and metoprolol) should be preferred to nonselective β-blockers in patients with COPD. Other drugs commonly prescribed in patients with CVD are antiplatelet agents, statins, angiotensin conversion enzyme inhibitors (ACE-Is), and angiotensin receptor blockers (ARBs) [27]. Studies on the safety of these drugs in patients with COPD have been conducted. Antiplatelet therapy has been associated with a 1-year reduction in mortality in patients hospitalized for an acute exacerbation of COPD [28,29]. A lot of patients with COPD take statins for the primary or secondary prevention of CVD. Statins reduce cholesterol and also have antioxidant, anti-inflammatory, and immunomodulatory effects [30]. Several observational studies have found an association between statin use and fewer exacerbations, and lower hospitalization and mortality rates in COPD patients [31,32]. However, randomized controlled trials (RCTs) have failed to confirm any such associations [33]. The ACE Inhibitor/ARB association could be a treatment option to increase survival chances and reduce the risk of severe exacerbations in COPD patients [34]. An analysis of the Multi Ethnic Study of Atherosclerosis (MESA) has suggested that these drugs could reduce the progression of emphysema through the inhibition of transforming growth factor (TGF)-β signaling [35]. The most common side effect of ACE-Is is coughing, which may be problematic for patients with COPD.

### 2.4. Summary of Evidence and Future Perspective

In conclusion, the biological and mechanic consequences of COPD affect cardiovascular hemodynamics and the risk of major CV events. While it is well established that COPD-associated hypoxemia is responsible for group III pulmonary hypertension, and at this time, no current treatment has been approved as previous trials documented contrasting results. A clinical trial investigating the role of sildenafil in modifying exercise tolerance and shortness of breath in COPD is currently ongoing (NCT05061368). Endothelial dysfunction and vascular perfusion will be also evaluated in a trial (NCT05567562) testing the efficacy of dual antiplatelet treatment in changing the pulmonary perfusion measured with contrasted computed tomography. Furthermore, the above-reported literature suggests the unremarkable role of bronchodilators in improving left ventricle hemodynamics; the clinical trial NCT04522596 will add novel data about the diastolic function of the left ventricle in COPD patients treated with the combination of UME-VIL. Recently, two large RCTs have suggested a potential effect on CV mortality for triple inhaled therapy, although some criticisms have been postulated, and real-life data are still missing. Novel data from large observational studies will add more information to this field (NCT05652439). For patients with CV diseases or risk factors, despite a certain reappraisal of cardioselective b-blockers, some uncertainties about their use in clinical practice still exist. NCT03917914 is an ongoing phase III trial testing the safety and efficacy of bisoprolol versus a placebo in patients with CV risk factors and concurrent COPD. Likewise, in the same respective, a large interventional phase IV study on metoprolol (NCT03566667) has been planned. Finally, as contrasting results about statins in preventing COPD acute exacerbations still exist, an ongoing phase IV trial of atorvastatin versus placebo is currently ongoing, and the data are expected to be published by 2026.

## 3. COPD and Lung Cancer

With an estimated 1.6 million deaths each year, lung cancer remains the deadliest cancer worldwide. Various epidemiological and observational study have extensively confirmed the association between LC and COPD [36]. In this respect, sharing risk factors play a crucial role in susceptible individuals. Tobacco exposure stands undoubtedly as a leading common denominator shared by the two diseases; however, it must be considered that only about 30% of smokers eventually develop clinically significant COPD, while 10–15% develop lung cancer [37]. In a similar manner, occupational as well as particulate matter (PM) exposure represent major risk factors for both LC and COPD [38,39,40,41]. Moreover, the scenario is by far more complicated. Chronic inflammation, genetic susceptibility, premature aging, as well as aberrant matrix remodeling are only few of the potential common pathogenetic pathways. With regard to genetic predisposition, published genome-wide association studies of lung cancer and COPD have identified overlapping chromosomal regions and genes, such as chromosome 15q24/15q25.1 and chromosome 4q22. The former includes the nicotinic acetylcholine receptor A subunits 3 and 5 (CHRNA3 and CHRNA5) genes, while the latter contains the FAM13A gene, coding for the N-terminal extension containing the Rho-GAP domain which presents tumor suppression activity through the inhibition of the intracellular signal transduction molecule RhoA [42,43]. Moreover, the 4q31 locus, containing the Hedgehog interacting protein (HHIP) gene, which mediates epithelial mesenchymal transformation (EMT) due to smoking, has been reported to have a protective effect on both LC and COPD, particularly rs1489759 and rs2202507 single-nucleotide polymorphisms (SNPs). Conversely, rs7689420, rs1489758, rs1489759, and rs10519717 SNPs were associated with the occurrence of LC among COPD patients [44,45]. In addition to genetic predisposition, epigenetic alterations such as DNA methylation, histone modification, and miRNA expression represent other major factors involved in gene expression in COPD subjects, increasing their susceptibility to develop LC. An epigenome-wide association study has showed an increased methylation and the subsequent down-regulation of coiled-coil-domain-containing 37 (CCDC) and microtubule-associated protein 1B (MAP1B) genes, leading to augmented cell proliferation and lung cancer risk [46]. Due to the biological process by which epithelial cells produce phenotypic and structural changes leading to a distinct mesenchymal phenotype, EMT has been regarded with interest as the origin of LC among COPD patients. In this regard, a high level of positive airway epithelial mesenchymal markers and vascular proliferation as well as a reduction in core epithelial markers in airways of COPD patients has been observed. Specifically, EMT-3 is closely associated with angiogenesis and most lung squamous cell carcinoma [47]. Cigarette smoke and oxidative stress cause damage to epithelial cells, leading to apoptosis and emphysema and, on the other hand, promote the expression of hypoxia-inducible factor-1 α (HIF-1) and vascular endothelial growth factor (VEGF), accelerating the proliferation and invasion of tumors [48]. Likewise, smoke exposure jeopardizes the normal lung barrier function, leading to chronic inflammation to sustain the progressive acquisition of fibroblast-related markers, such as vimentin, type I collagen, and smooth muscle actin, by epithelial cells, a process known as endothelial mesenchymal transformation (EndoMT) [49]. In light of its involvement in chemotherapy and radiation resistance, EndoMT has been regarded as an attractive therapeutic target for increasing the efficacy of therapies [50].

The tumor microenvironment (TME) represents a heterogeneous milieu composed of different cellular components, growth factors, proteases, and the extracellular matrix (ECM) in which a tumor itself progresses. Leukotrienes, which are pro-inflammatory lipid mediators mainly produced by mast cells, macrophages, neutrophils, and eosinophils, are critical component of TME and have been found to be increased in COPD patients. With respect to the ECM, the aberrant expression of matrix metalloproteinases (MMPs), such as MMP-2 and MMP-9, over tissue inhibitors of specific metalloproteinases (TIMPs) has been demonstrated to be associated with both COPD and tumor cell proliferation [48,51]. In patients affected by COPD, a pro-tumor inflammatory microenvironment is known to promote an uncontrolled proliferation and inhibit apoptosis. Tumor associated macrophages (TAMs) represent crucial elements of lung TME. It has been fairly demonstrated that TAMs may polarize into M1 or M2 phenotypes, exhibiting both antitumor and pro-tumor bidirectional effects, respectively. By secreting pro-inflammatory cytokines and chemokines and promoting the Th1 reaction, M1-type macrophages show positive immune response. On the other hand, M2 macrophages show a low aptitude for antigen presentation and can prompt a Th2 response by releasing inhibitory cytokines such as IL-10 or TGF-β. As a result, the immune response is turned down, and tissue repair as well as angiogenesis are promoted, leading to the tumor being sustained [52,53,54]. Interestingly, in alveolar regions as well as in the airways of COPD patients, M2 macrophages predominate due to the abundance of Th2 cytokines including IL-4, IL-10, IL-13, CCL22, and IL-6 [52]. From a clinical perspective, COPD may negatively affect lung cancer prognosis [55]. In a cohort of 1126 patients with lung cancer undergoing surgical resection, patients with COPD had a significantly higher incidence of post-surgical complications, in particular, pneumonia and prolonged air leak, than controls, although the presence of COPD itself did not impact patients’ long-term survival [56]. However, Bugge et al. have reported that patients with severe COPD showed a significantly reduced cumulative survival after 2 and 5 years of 63.5% (95% (CI), 48.4% to 75.2%) versus 81.7% (95% CI, 77.4% to 85.2%) and 41.8% (95% CI, 26.5% to 56.3%) versus 61.3% (95% CI, 55.3% to 66.6%), respectively [57]. In advanced LC stages, immune checkpoints inhibitors (ICIs) have revolutionized the therapeutic landscape [58,59,60]. In advanced stages of NSCLC, the tumor proportion score (TPS) of the PD-L1 on biopsy emerged as an important response biomarker [61]. Increased PD-L1 expression is associated with cigarette smoke exposure. Furthermore, in GOLD stages 1–2, COPD patients have a higher PD-L1 expression when compared to controls or more advanced stages (GOLD 3–4) [62]. Data indicate that COPD patients may benefit from immunotherapy more than non-COPD subjects [63]; however, the impact of COPD has also been reported to be negative in terms of both survival and tolerability in LC patients treated with ICIs. As reported by Zhang and coworkers, the incidence of immune-related adverse events (irAEs) in patients with no COPD and/or mild to moderate COPD was significantly lower than that in patients with severe COPD (*p* = 0.003). In addition, patients with mild to moderate COPD have a higher objective response rate (ORR), disease control rate (DCR), and cytokine (IL-6, IL-8, and IL-10) levels compared with the severe COPD group [64]. Conversely, in a cohort of 156 patients, patients with COPD have a significantly longer progression-free survival (PFS) (*p* = 0.018) [65]. In this regard, the evidence supports the presence of several distinct sub-phenotypes of COPD with various immune profiles with different potential response to ICIs [66,67]. Likewise, an increased mutational burden due to prolonged carcinogen exposure may in part explain the improved response to ICI treatment of LC patients with COPD [68].

### Summary of Evidence and Future Perspective

COPD and LC are two highly prevalent disorders with a noteworthy impact in terms of clinical and economic burden. Strategies tackling early LC detection among COPD patients are still heterogeneous despite the result of recent LC screening trials. At the same time, currently ongoing LC screening programs may offer the chance to promote early COPD intervention and smoking cessation for subjects involved in the trial (NCT05766046; NCT05444062). Sharing a molecular pathogenesis may constitute ideal targets for personalized strategies to reduce the burden of both diseases. In early LC stages, it has been established that COPD therapeutic optimization may ameliorate surgical outcomes. Similarly, several studies have suggested that patients with advanced-stage LC and concurrent COPD, when treated with anti-PD-1/PD-L1 therapies, might have greater benefits from these agents in terms of overall survival (OS), PFS, and ORR while also improving the clinical and functional outcomes of COPD—including FEV1 and FVC. Conversely, data on IRAEs in more advanced COPD stages should be considered in clinical practice while balancing the potential advantages and potential side effects. Data from a recently completed phase II clinical trial of pembrolizumab in NSCLC and COPD patients are anticipated (NCT05578222). The profound knowledge about the interaction between this pharmacological intervention and the tumor immune microenvironment may offer novel opportunities for targeting these complex disorders.

## 4. COPD and Bronchiectasis

Bronchiectasis is a chronic airway disease, in which bronchi become progressively and permanently dilated as a result of a vicious circle involving the inflammation, infection, and repair of the bronchial mucosa, leading to lesions in the mucociliary system and mucus retention [69]. Bronchiectasis affects approximately 30% of patients with COPD, but the prevalence is higher in advanced stages [70,71,72]. Chest high-resolution computed tomography (HRCT) constitutes the gold standard for the diagnosis of bronchiectasis. In recent years, HRCT has been more commonly used on patients with COPD (e.g., assessment for volume reduction, screening for lung cancer, and follow-up on nodules), so the abnormalities of the bronchial tree has been increasingly observed. In patients with COPD, bronchiectasis is mainly cylindrical (70–90%), predominantly in the lower lobes of the lung (52–81%), bilateral (52–67%), and results in moderate scores in radiological analyses of severity [73,74,75]. Chronic bronchial infection is often detected in patients with COPD, and there may be a connection between the two conditions [71]. COPD patients with bronchiectasis have increased sputum production and purulence and frequently experience the colonization of the bronchial mucosa due to potentially pathogenic microorganisms (PPMs), particularly *Pseudomonas aeruginosa*, and experience more frequent and severe exacerbations [76]. A meta-analysis by Du et al. showed that patients with bronchiectasis and COPD have a significant increase in mortality risk [77]. In line with these results, De Soyza et al. demonstrated that the frequency of hospitalization, exacerbation rates, lung function severity, and ambulatory visits are increased in patients with bronchiectasis and COPD overlap (BCO) compared to patients with bronchiectasis only [78]. Dou et al. demonstrated that COPD patients with concomitant bronchiectasis presented with worse pulmonary function (FEV1% predicted), a higher emphysema index (EI) score, and a higher proportion of pulmonary hypertension and cor pulmonale than patients without bronchiectasis [79].

As result of chronic bronchial infection and consequent inflammation in both COPD and bronchiectasis, serum levels of inflammatory biomarkers, such as IL-6, IL-8 and CRP are increased in BCO patients [80]. Also, Nigro et al. showed that in BCO patients, the levels of inflammatory cytokines such as IL-2, IL-4, IL-8, IFN-γ, and GM-CSF were significantly higher, while IL-10 expression levels were lower than those of the control group. The authors also investigated the serum level of adiponectin, a new biomarker in COPD, and they found a further increase in the levels of adiponectin in patients with BCO, which is probably related to their worse airway inflammatory state [81]. This assumption implies that adiponectin could be a potential biomarker to identify severe COPD patients with bronchiectasis [82].

Regarding therapy, long-term therapy with macrolides have been investigated in both bronchiectasis and COPD, but there are no studies specifically on BCO for either macrolide. Generally, there are significant concerns around the use of macrolides, including the adverse cardiovascular effects and antibiotic resistance. In addition, macrolides may increase the risk of treatment failure and mortality in nontuberculous mycobacterial infections (reported in up to 10% of COPD patients) [83,84]. Inhaled antibiotics are currently recommended in bronchiectasis but not in COPD patients owing to the considerable risk of bronchospasm, but some evidence suggests that selected COPD patients, such as those with bronchiectasis and/or with chronic *P. aeruginosa* infection, could benefit from these treatments [84]. Finally, inhaled steroids (ICS) are used on COPD patients with multiple exacerbations or peripheral eosinophilia. In regard to bronchiectasis, there is little evidence of the benefit of ICSs, probably due to the neutrophilic inflammation observed in this disease and the possible risk that they may generate more infections due to their immunosuppressive properties [85]. However, recent evidence suggests that for BCO patients with peripheral blood eosinophilia, using inhaled corticosteroids may have an impact on exacerbations [86].

### Summary of Evidence and Future Perspective

Hypersecretion and low tract respiratory infections often represent clinical hallmarks in patients affected by either COPD or bronchiectasis as well as BCO. It has been reported that airway mucus hypersecretion is associated with an accelerated lung function decline, higher rate of hospitalization and bacterial and viral mediated acute exacerbation in patients affected by COPD [78]. The use of ICS has long been debated in patients with bronchiectasis and COPD due to the risk of superimposed infections or bacterial colonization. However, recent body of the literature suggests that using peripheral eosinophilia a biomarker-based approach might be reasonable to select BCO patients who may have a benefit in reduction in acute exacerbation. While at this time no standard treatment have been approved for bronchiectasis alone or in overlap with COPD, the expiratory flow accelerator (EFA) has lately emerged as a valid technology in patient affected by COPD for improving exercise capacity as well as pulmonary function [79]. In this respect, a trial on impact of EFA in patients with COPD and bronchiectasis has been currently ongoing (NCT06017739).

## 5. COPD and OSA

Obstructive sleep apnea (OSA) is a disease characterized by a complete cessation (apnea) of or decrease (hypopnea) in airflow during sleep due to recurrent episodes of upper airway collapse, causing nocturnal oxygen desaturations, sleep fragmentation, and daytime sleepiness [87]. It is a relatively common disease which is often underestimated, even though it impacts one’s quality of life. The 2018 American Thoracic Society Research Statement highlighted the importance of focusing on sleep as a relevant factor in the care of patient with COPD [88]. The coexistence of OSA and COPD is known as overlap syndrome (OS) and was first described in 1985 by Flenley [89], who speculated the potential synergy between the two conditions. Epidemiological studies show that the prevalence of OS is 1–3.6% in the general population [90].

Interestingly, OS patients have an increased risk of acute exacerbations and mortality compared to patients with either COPD or OSA alone [91]. For this reason, the 2018 American Thoracic Society Research Statement highlighted the importance of focusing on sleep as a relevant factor in the care of patients with COPD [88].

During sleep, many changes in breathing occur; in particular, there is a reduction in respiratory muscle activity and diminished respiratory drive [92]. Likewise, in a cohort of 380 patients hospitalized due to COPD exacerbation, it has been demonstrated that 46% of subject have been found to have OSA. Noteworthily, readmission rates were significantly higher at 30, 60, and 90 days for subjects with OSA compared to patients without sleep disorders. In addition, mortality was significantly lower in patients without OSA [93].

Changes in lung mechanics lead to ventilation–perfusion mismatch and decreased functional residual capacity. During REM sleep, the activity of accessory respiratory muscles and intercostal muscles stops, and ventilation occurs due to diaphragm contraction. Although there are no consequences in normal subjects, COPD patients may be unable to tolerate these changes. The physiological compensatory response is represented by an increased respiratory rate or reduced expiratory time: these responses, in COPD patients, lead to increased hyperinflation and breathing effort [94]. In addition, when sleeping in the supine position, the upper airway resistance increases, leading to airflow obstruction, which may worsen the hyperinflation and hypoventilation in COPD patients [95]. Factors that facilitate respiratory obstructive events in COPD patients include rostral fluid, shifts from the supine position, and smoking-related upper airway inflammation [96,97]. As a result, COPD patients have poor sleep quality, frequent awakenings, and nocturnal oxygen desaturations, which have important clinical consequences, such as increased risk of cardiovascular events, hospitalizations, and mortality [98].

Chaouat et al. showed that PaO2 levels were lower, while PaCO2 levels were higher in OS patients compared to OSA patients [99]. In OS, episodic sleep desaturation occurs due to a low saturation baseline, resulting in intermittent hypoxia that worsen the already hypoxic state of the patient between episodes [100]. This results in the release of inflammatory mediators, oxidative stress, and an increased sympathetic tone which lead to endothelial dysfunction and the increased risk of CVDs [101]. Furthermore, hypoxemia in OS is more prolonged, so these patients have a higher incidence of pulmonary hypertension than COPD or OSA alone [102]. While continuous positive airway pressure (CPAP) is a well-recognized therapy for moderate and severe OSA [103], data regarding the effect of CPAP treatment on OS are limited since randomized clinical trials have not been performed yet; however, a cohort study showed that exacerbation rates and mortality are reduced in OS patients treated with CPAP [91]. Interestingly, when OS patients have severe emphysema with hypoventilation, non-invasive ventilation in BiPAP mode is more effective than CPAP in increasing minute ventilation and reducing hyperinflation and breathing effort [104]. Finally, OS patients may still have low oxygen saturation levels despite the use of CPAP. In these cases, supplemental oxygen therapy during sleep is indicated to improve quality of life [100].

### Summary of Evidence and Perspective

Several mechanisms are shared between COPD and sleep-disordered breathing (SDB), and a bidirectional relationship has been demonstrated. The magnitude of diagnosing and treating SDB—particularly OSA and OS—on COPD clinical outcomes has recently emphasized in the recent literature and international statements. An interventional clinical trial is currently ongoing to evaluate the impact of the early diagnosis of OSA and treatment with CPAP on the hospital readmission of hospitalized patients with COPD (NCT03647462). Likewise, insomnia, a condition characterized by a dissatisfaction in terms of either sleep duration or sleep quality, represents a potential concurrent disorder in patient with OSA, COPD, and OS. Primarily related to tobacco use or anxiety, insomnia undoubtedly has a deleterious impact on both cardiovascular risk and quality of life [105,106,107]. The management of insomnia in patients with COPD or OSA is extremely challenging due to risks of serious adverse effects. In this regard, cognitive behavior therapy represents the first-line treatment for these patients [108,109]. Lately, lemborexant, a dual orexin receptor antagonist, has recently been demonstrated respiratory safety in both COPD [110] and OSA (NCT04647383) [111] cohorts, opening a new perspective in the treatment of this treatable trait among these patients.

## 6. COPD and Metabolic Syndrome

Almost more than 30% of COPD patients present one or more components of Metabolic Syndrome (MetS), including abdominal obesity, dyslipidemia, hypertension, and hyperglycemia, worsening the prognosis [112]. COPD patients with MetS are more frequently females, have higher BMI and higher FEV1 scores than COPD patients without MetS [113]. The major prevalence of MetS in patients with milder airflow obstruction reflects the weight loss noted in severe COPD and the high cardiovascular-related mortality of COPD patients with MetS, so they may die earlier due to CVD mortality, thus not reaching end-stage COPD [112,114]. Several studies reported that patients with COPD and a lower BMI score have an increased mortality risk in comparison with overweight or obese patients; this is known as “the obesity paradox”. A possible reason is that the BMI cannot differentiate between metabolically and functionally active lean mass (muscle) and fat mass, and the majority of patients with COPD have a progressive loss of muscle mass. Another possible explanation is that the decreased lung volumes in obese patients may defend against hyperinflation in advanced COPD, improving lung function [115]. The development of MetS in COPD is multifactorial, but oxidative stress, systemic inflammation, and decreased physical activity are common features of both diseases [116]. As mentioned above, lung inflammation during COPD leads to a chronic increase in pro-inflammatory cytokines such as IL-6, IL-1β, CRP, and TNF -α at both the serum and airway level [117]. These pro-inflammatory cytokines promote insulin resistance, which in turns favors the development of diabetes mellitus type 2. Also the oxidative stress caused by cigarette smoke or by the host’s inflammatory response decreases insulin resistance [118]. systemic chronic inflammation also promotes the formation of atherosclerotic plaques. A vicious cycle linking MetS and COPD have been postulated: the deterioration of the lung function in COPD patients leads to physical inactivity, increasing the tendency for weight gain, and excess weight gain not only accelerates the decline of lung function but it also puts further restrictions on physical activity [119]. These patients present with more severe dyspnea and less exercise tolerance in comparison with non-obese patients with COPD [120]. Moreover, specific factors related to COPD, such as steroid use, may also contribute to MetS. Oral glucocorticoids, used by patients experiencing acute exacerbations of COPD (AECOPD), can increase blood glucose levels, HDL levels, and appetite, and cause muscle atrophy and abdominal obesity. Adipose tissue inflammation has been suggested as another key factor interconnecting COPD and MetS. Adipose tissue is an organ with an active role in the production and release of several proteins, such as adiponectin, with various physiological functions, such as immunity, insulin sensitivity, lipid and glucose metabolism, and inflammation [121,122,123]. In obese patients, serum levels of total adiponectin and its isoforms are reduced in a disproportionate way [81]. Also, leptin is produced by adipose tissue, and it is implicated in the regulation of energy balance and food intake in a feedback mechanism involving the hypothalamus. Leptin also has an immunomodulatory function, leading to the overexpression of pro-inflammatory cytokines. The expression of leptin receptors in the lung suggests that this mediator could act also on the lung. Nicotine promotes the production of leptin from adipose tissue. On this note, augmented systemic concentrations of leptin are associated with a decline in pulmonary function, and during AECOPD, levels of serum leptin and the ratio of leptin to adiponectin seem to increase [124].

Patients with concomitant COPD and MetS may be considered “high-risk” and should be closely monitored. Metformin is the recommended first-line treatment for type 2 diabetes mellitus, and it has anti-inflammatory and antioxidative properties, giving a possible adjunctive benefit in the prevention and treatment of respiratory diseases. Conditions that may cause tissue hypoxia or metabolic acidosis are contraindications to metformin, but unless respiratory failure is present, COPD is not a contraindication to metformin therapy. Sexton et al. showed that metformin had a positive effect on dyspnea and respiratory muscle strength in COPD patients with diabetes [125].

On the contrary, Roflumilast, a Phosphodiesterase-4 inhibitor, may be considered in patients with COPD and persistent exacerbations and an FEV1 < 50% of predicted and chronic bronchitis. A study showed that Roflumilast could induce a reduction in body fat mass in patients with COPD while also modulating insulin sensitivity [126].

Regarding the therapies addressing oxidative stress, flavonoid and polyphenol supplementation, such as resveratrol, has proved to be powerful in MetS and COPD patients [127]. Finally, the “obesity paradox” has been put into question regarding the suitability of targeting obesity in COPD patients because the weight loss results not only in reduced fat mass but also in the depletion of muscle mass. The GLP-1 receptor agonist (GLP-1RA) liraglutide has been approved for weight loss in patients with obesity. A study by Dogan et al. suggested the use of liraglutide in patients suffering from COPD and obesity for the additional anti-inflammatory actions of GLP-1RAs. The authors found that liraglutide use improved forced vital capacity (FVC), carbon monoxide diffusion capacity, and CAT-scores, and it also induced significant weight loss [128].

### Summary of Evidence and Perspective

Altogether these data suggest that low-grade systemic inflammation and adipose-tissue-related hormones are the main players influencing the onset and clinical behavior of COPD and MetS. Hypoglycemic agents are currently observed with interest based on their pleiotropic effect for possible implication on COPD outcomes. As MetS can be attenuated with diet and physical exercise many programs are currently ongoing to evaluate the efficacy on patient-reported outcomes among COPD patients. NCT05806294 is a Canadian phase IV study that is integrating a dietary and physical exercise program using digital technology directed in patients with concurrent COPD and MetS.

## 7. Conclusions

There is now a growing interest in the detrimental impact of comorbidities in patients affected by COPD due to their significant clinical and economic implications. Comorbidities make the management of COPD difficult, and greater attention needs to be paid in both clinical and research settings. The underlying mechanisms linking COPD with its comorbidities may constitute ideal targets for personalized strategies.

The mounting evidence from RCTs indicates that COPD treatment might influence cardiovascular mortality; the magnitude of this effect needs further validation in real-life scenarios. The role of cardiovascular agents—b-blockers, ACE-i/ARBs, statins, and antiplatelets—in influencing COPD outcomes has not been definitively ruled out, and ongoing studies will add informative data. Similarly, lung cancer treatments in mild to moderate COPD patients seems to be potentially synergic, tackling both oncological and respiratory outcomes, while patients in more advanced COPD stages have a higher risk of adverse events.

Finally, novel data supporting the efficacy of non-pharmacological strategies including EFA in patients with bronchiectasis and COPD, cognitive behavior therapy for the management of insomnia in patients with COPD and OSA, and diet and physical exercise in patients with COPD and MetS are anticipated. In conclusion, the analysis of COPD comorbidities may offer the opportunity to select an approach based on treatable traits, prompting a pragmatical and holistic therapy approach in COPD patients.

## Data Availability

Not applicable.
